# Virulence factors and antibiotic resistance of *Helicobacter pylori* isolated from raw milk and unpasteurized dairy products in Iran

**DOI:** 10.1186/1678-9199-20-51

**Published:** 2014-12-04

**Authors:** Soolmaz Mousavi, Farhad Safarpoor Dehkordi, Ebrahim Rahimi

**Affiliations:** Young Researches and Elites Club, Shahrekord Branch, Islamic Azad University, Shahrekord, Iran; Department of Food Hygiene, College of Veterinary Medicine, Islamic Azad University, Shahrekord Branch, PO Box 166, Shahrekord, Iran

**Keywords:** *Helicobacter pylori*, Virulence factors, Antibiotic resistance properties, Milk, Dairy products, Iran

## Abstract

**Background:**

Despite the high importance of *Helicobacter pylori*, the origin and transmission of this bacterium has not been clearly determined. According to controversial theories and results of previous studies, animal source foods – especially milk – play an important role in the transmission of *H. pylori* to humans. The aim of the present study was to determine the distribution of *vacA*, *cagA*, *iceA* and *oipA* virulence factors in *H. pylori* strains isolated from milk and dairy products and study their antimicrobial resistance properties.

**Methods:**

A total of 520 raw milk and 400 traditional dairy product samples were cultured and tested. Those that were *H. pylori*-positive were analyzed for the presence of *vacA*, *cagA*, *iceA* and *oipA* virulence factors. Antimicrobial susceptibility testing was performed by the disk diffusion method.

**Results:**

One hundred and three out of 520 milk samples (19.8%) and 77 out of 400 dairy products samples (19.2%) were contaminated with *H. pylori*. The most frequently contaminated samples were ovine milk (35%) and traditional cheese (30%). Total prevalence of *vacA*, *cagA*, *iceA* and *oipA* factors were 75%, 76.6%, 41.6% and 25%, respectively. *H. pylori* strains of milk and dairy products harbored high levels of resistance to ampicillin (84.4%), tetracycline (76.6%), erythromycin (70.5%) and metronidazole (70%).

**Conclusions:**

High presence of antibiotic-resistant strains of *H. pylori* suggest that milk and dairy samples may be the sources of bacteria that can cause severe infection. Our findings should raise awareness about antibiotic resistance in *H. pylori* strains in Iran.

## Background

Milk plays an important role in the nutrition of Iranian people since it is considered a complete food source, particularly for children and the elderly. Milk of animal origin is usually manufactured into more stable dairy products of worldwide commerce such as yogurt, cream, butter and cheese. Every day millions of people consume milk and dairy products. Therefore, hygienic quality of produced milk is extremely important regarding public health hazards.

*Helicobacter pylori* is a microaerophilic gram-negative bacterium with a curved spiral shape which is known as the causative agent of type B gastritis, peptic ulcer disease, gastric adenocarcinoma and mucosa-associated lymphoid tissue lymphoma [1]. In spite of the general idea about the low prevalence of gastric cancer, it is considered the fourth most common type of cancer and the second leading cause of cancer-related deaths worldwide [2]. A total of 1,665,540 new cancer cases and 585,720 cancer deaths are estimated to occur in the United States in 2014 [3].

Several studies have indicated the presence of *H. pylori* in the stomach of domestic animals in the absence of gastritis [4–7]. It was also isolated in milk of sheep, goat, cow, buffalo and camel species. These findings indicate that domestic ruminants may be a natural host for *H. pylori.* Moreover, the detection of *H. pylori* in the milk and feces of domestic animals suggests that *H. pylori* may be regarded as a zoonotic infection [4–7].

Good conditions for the survival of *H. pylori* in animal milk provides opportunities for its transmission to humans [8]. At temperatures below 30°C, *H. pylori* is capable of surviving in milk and water, fresh fruit and vegetables, fresh meat (including red meat, poultry and fish) and some dairy products [8].

Data on the epidemiology and transmission of *H. pylori* is extremely significant in order to prevent its distribution and to identify high-risk populations, especially in areas that have high rates of gastritis, peptic ulcers, and gastric cancer such as Iran [5–7]. In addition to routes of transmission, treatment is a critical point in the epidemiology of *H. pylori* infection in humans, since therapeutic options have become somewhat limited because of the presence of multidrug resistant strains of this bacterium [9]. Moreover, to the best of our knowledge, we could not find any published data on the antibiotic resistance pattern of *H. pylori* strains isolated from animal source foods.

Another aspect of *H. pylori* epidemiology, connected to its pathogenicity, is related to putative virulence factors. In fact, to determine the pathogenicity of *H. pylori*, assessment of latent virulence factors is required. Survival and success of pathogens require that they colonize the host, reach an appropriate niche, avoid host defenses, replicate, and exit the infected host to spread to an uninfected one. In *H. pylori*, the genes that are mostly associated with infections include cytotoxin-associated gene A (*cagA*), vacuolating cytotoxin (*vacA*), outer inflammatory protein (*oipA*) and finally the gene induced by contact with epithelium (*iceA*) [10, 11]. These genes are usually related to adhesion to gastric epithelial cells [10, 11].

It has been reported that the *cagA* gene is present in about 60% of *H. pylori* isolates from clinical samples. This gene is associated with inflammation by activation of NF-kB and secretion of cytokines and chemokines such as interleukin 8 (IL-8) [10, 11]. The gene *vacA* plays a possible role on the formation anion-selective channels within artificial membranes and it is assumed to do the same *in vivo*, increasing absorbency to anions and urea [10, 11]. Endocytosis of *vacA* channels leads to the formation of large vacuoles within the late endosome-lysosome cubicle [10, 11]. The function of *iceA* gene is not yet clear. However, it is hypothesized that this gene is upregulated upon contact of *H. pylori* with the gastric epithelium and has been regarded as a marker for peptic ulcer diseases [12, 13]. Finally, *oipA* induces IL-8 secretion by epithelial cells and increases inflammation as well as the clinically important presentation of peptic ulcer [12, 13]. Considering the unclear epidemiological aspects of *H. pylori* infection, the present investigation was carried out in order to study the distribution of virulence factors and antibiotic resistance properties of *H. pylori* isolated from animal milk and dairy products.

## Methods

### Samples

Overall, 520 raw milk samples were collected from the following species: bovine (n = 120), caprine (n = 100), ovine (n = 100), buffalo (n = 80), camel (n = 60) and donkey (n = 60). The samples were obtained from farm bulk tanks and milk collection centers from several geographic regions of Iran, from March 2013 to March 2014. Bovine and buffalo samples were collected throughout this period. Because of the seasonality of their lactating periods, caprine, donkey and ovine milk samples were only available in certain months (from March through May and September to November in Iran). At each site, sampling was performed according to the International Dairy Federation guidelines [14]. Samples (100 mL, in sterile glass containers) were transported to the laboratory at 4°C within a maximum of 6 to 12 hours after collection.

For dairy products, 100 samples of cheese, 100 of butter, 100 of cream and 100 of ice cream made of raw milk were purchased from traditional supermarkets. All selected dairy products were made from unpasteurized milk and after collection were kept under refrigeration in plastic bags; information about date of production and shelf life were not available. These products are packed manually in traditional conditions and are popular among Iranian people because of their pleasant taste and smell. Dairy product samples were collected over a period of eight months (between August 2013 and February 2014), and were analyzed on the day of acquisition. Samples were transported under refrigeration (4-6°C) in thermal boxes containing ice packs and were tested immediately after collection.

### Isolation of *Helicobacter pylori*

Twenty five milliliter or grams of each sample were added to 225 mL of Wilkins Chalgren anaerobe broth (Oxoid, UK) supplemented with 5% of horse serum (Sigma, USA) and colistin methanesulfonate (30 mg/L), cycloheximide (100 mg/L), nalidixic acid (30 mg/L), trimethoprim (30 mg/L), and vancomycin (10 mg/L) (Sigma, USA) and incubated for seven days at 37°C under microaerophilic conditions (86% N_2_, 9% CO_2_, 5% O_2_) using the Bio-Bags (type Cfj; Becton Dickinson). Then, 0.1 mL of the enrichment selective broth was plated onto Wilkins Chalgren anaerobe agar (Oxoid, UK) supplemented with 5% of defibrinated horse blood and 30 mg/L colistin methanesulfonate, 100 mg/L cycloheximide, 30 mg/L nalidixic acid, 30 mg/L trimethoprim, and 10 mg/L vancomycin (Sigma, USA) [15] and incubated for seven days at 37°C under microaerophilic conditions. Suspected colonies were identified as *H. pylori* based on the morphology of the colonies, Gram stain, and production of oxidase, catalase, and urease [16]. The isolates were identified as *H. pylori* by using conventional bacteriological methods, and were also positive by the PCR assay. For comparison, a reference strain of *H. pylori* (ATCC 43504) was employed.

### Antimicrobial susceptibility testing

Pure cultures of *H. pylori* isolates, one strain from each positive sample, were used for antimicrobial susceptibility testing. Tests were performed by the Kirby-Bauer disc diffusion method using Mueller-Hinton agar (HiMedia Laboratories, India) supplemented with 5% defibrinated sheep blood and 7% fetal calf serum, according to the Clinical and Laboratory Standards Institute guidelines (CLSI) [17]. The antimicrobial resistance of *H. pylori* was measured against the widely used antibiotics in cases of *H. pylori* gastric ulcer. The following antimicrobial impregnated disks (HiMedia Laboratories, India) were used: metronidazole (5 μg), ampicillin (10 μg), clarithromycin (2 μg), erythromycin (5 μg), tetracycline (30 μg), amoxicillin (10 μg), levofloxacin (5 μg), trimethoprim (25 μg), cefsulodin (30 μg), and furazolidone (1 μg). After incubation at 37°C for 48 hours in a microaerophilic atmosphere, the susceptibility of the *H. pylori* to each antimicrobial agent was measured and the results were interpreted in accordance with criteria provided by CLSI [17]. *H. pylori* ATCC 43504 was used as quality control organism in antimicrobial susceptibility determination.

### DNA extraction and *Helicobacter pylori*16S rRNA gene amplification

Suspected colonies were identified as *H. pylori* though PCR technique. Genomic DNA was extracted from *H. pylori* colonies using a DNA extraction kit for cells and tissues (Roche Applied Science, Germany, 11814770001) according to the manufacturer’s instructions. Density of extracted DNA was assessed by optic densitometry. Extracted DNA was amplified for the 16S rRNA gene (primers: HP-F: 5'-CTGGAGAGACTAAGCCCTCC-3' and HP-R: 5'-ATTACTGACGCTGATTGTGC-3') [18]. PCR reactions were performed in a final volume of 50 μL containing 5 μL 10 × buffer + MgCl_2_, 2 mM Dntp (Fermentas, Germany), 2 unit Taq DNA polymerase (Fermentas, Germany), 100 ng genomic DNA as a template, and 25 picomole of each primer (Cinagen, Iran). PCR was performed using a thermal cycler (Flexcycler^2^ Gradiant, Eppendorf, Germany) under the following conditions: an initial denaturation of two minutes at 94°C; 30 cycles of 95°C for 30 seconds, 60°C for 30 seconds, 72°C for 30 seconds and a final extension at 72°C for eight minutes.

### Virulence factors of *Helicobacter pylori*

The list of primers used for detection of *vacA*, *cagA*, *oipA* and *iceA* virulence factors in *H. pylori* is shown in Table 1. PCR was performed in a total volume of 50 μL that contained 1 μM of each primer, 1 μL of genomic DNA (approximately 200 ng), 1 mM dNTPs mix (Fermentas, Germany), 2 mM MgCl_2_, and 0.05 of U/μL Taq DNA polymerase (Fermentas, Germany). PCR amplifications were performed in an automated thermal cycler (Mastercycler Gradient, Eppendorf, Germany). The following cycle conditions were used for PCR amplification: for *vacA* – 35 cycles of 30 seconds at 95°C, 60 seconds at 66°C, and five minues at 72°C; for *cagA* – 35 cycles of 30 seconds at 95°C, 60 seconds at 55°C, and five minutes at 72°C; for *iceA* – 35 cycles of 30 seconds at 95°C, 60 seconds at 56°C, and five minutes at 72°C; and, finally, for *oipA* – 60 seconds at 94°C, 60 seconds at 52°C and 60 seconds at 72°C. All runs included one negative DNA control consisting of PCR grade water and two or more positive controls (26695, J99, SS1, Tx30, 88–23, 84–183 and positive samples).

**Table 1:** **Oligonucleotide primers used for detection of** ***vacA*** **,** ***cagA*** **,** ***oipA*** **and** ***iceA*** **virulence factors of** ***Helicobacter pylori*** **strains isolated from milk and dairy products** [19, 20]

Gene name	Primer sequence (5’-3’)	Size of product (bp)
*vacA*	F: GCCGATATGCAAATGAGCCGC	678
	R: CAATCGTGTGGGTTCTGGAGC	
*cag A*	F: AATACACCAACGCCTCCAAG	400
	R: TTGTTGGCGCTTGCTCTC	
*iceA*	F: CGTTGGGTAAGCGTTACAGAATTT	557
	R: TCATTGTATATCCTATCATTACAAG	
*oip A*	F: GTTTTTGATGCATGGGATTT	401
	R: GTGCATCTCTTATGGCTTT	

PCR products were resolved by agarose gel electrophoresis (5 V/60 min) using 1.5% agarose in Tris Acetate-EDTA (TAE) buffer containing 0.5 μg/mL of ethidium bromide (Merck, Germany). Molecular size ladder of 100 bp (Fermentas, Germany) was used to determine the size of the bands. The gel was viewed and photographed on a Gel-Doc System (Bio-Rad, USA). All tests were performed in triplicate.

### Statistical analysis

Data were transferred to Microsoft Excel spreadsheet (Microsoft Corp., USA) for analysis. Using SPSS 16.0 statistical software (SPSS Inc., USA), chi-squared test and Fisher’s exact two-tailed test analysis were performed and differences were considered significant at values of *p* <0.05. Distribution of virulence factors and antimicrobial resistance properties of *H. pylori* isolated from the milk and dairy products were statistically analyzed.

## Results and discussion

### Prevalence of *Helicobacter pylori*in studied samples

Eight hundred and sixty milk and dairy samples were analyzed for the presence of *H. pylori*. Distribution of *H. pylori* in various types of milk and dairy products is shown in Table 2. Out of 920 milk and dairy samples, 180 (19.5%) were positive for *H. pylori*. Specifically, among the positive samples, 103 out of 520 were milk (19.8%) and 77 out of 400 consisted of dairy samples (19.2%). Ovine milk (35%) and traditional cheese (30%) were the most commonly contaminated products.

**Table 2:** **Total distribution of** ***Helicobacter pylori*** **in various types of milk and dairy products**

Type of samples	Number of samples	Occurrence of ***Helicobacter pylori*** (%)
Bovine raw milk	120	20 (16.6)
Ovine raw milk	100	35 (35)A*
Caprine raw milk	100	28 (28)
Buffalo raw milk	80	12 (15)a
Camel raw milk	60	8 (13.3)a
Donkey milk	60	–
**Total milk**	**520**	**103 (19.8)**
Traditional cheese	100	30 (30)B
Traditional cream	100	15 (15)
Traditional butter	100	5 (5)b
Traditional ice cream	100	27 (27)
**Total dairy products**	**400**	**77 (19.2)**
**Total**	**920**	**180 (19.5)**

*The same small and capital letters in columns indicate a significant difference about *p* <0.05.

### Distribution of virulence factors in *Helicobacter pylori*isolates

Distribution of putative virulence factors in *H. pylori* strains isolated from milk and dairy products is shown in Table 3. Of 180 isolated strains of *H. pylori*, the distribution of *vacA*, *cagA*, *iceA* and *oipA* virulence factors were 135 (75%), 138 (76.6%), 75 (41.6%) and 45 (25%), respectively. The most commonly detected virulence factor in *H. pylori* was *cagA*. Isolates from caprine milk and traditional cheese had the highest incidence of putative virulence genes.

**Table 3:** **Distribution of putative virulence factors in** ***Helicobacter pylori*** **strains isolated from various types of milk and dairy products**

Type of samples (n. positive)	Distribution of virulence factors (%)			
	***vacA***	***cagA***	***iceA***	***oipA***
Bovine raw milk (20)	15 (75)	14 (70)	10 (50)	8 (40)
Ovine raw milk (25)	22 (88)	23 (92)	12 (48)	5 (20)
Caprine raw milk (28)	25 (89.2)	27 (96.4)	14 (50)	10 (35.7)
Buffalo raw milk (12)	9 (75)	10 (83.3)	4 (33.3)	2 (16.6)
Camel raw milk (8)	5 (62.5)	5 (62.5)	2 (25)	1 (12.5)
Donkey raw milk (−)	–	–	–	–
**Total milk (103)**	**76 (73.7)**	79 (76.6)A*	**42 (40.7)**	**26 (25.2)a**
Traditional cheese (30)	26 (86.6)	26 (86.6)	15 (50)	8 (26.6)
Traditional cream (15)	10 (66.6)	11 (73.3)	7 (46.6)	4 (26.6)
Traditional butter (5)	3 (60)	3 (60)	1 (20)	–
Traditional ice cream (27)	20 (74)	19 (70.3)	10 (37)	7 (25.9)
**Total dairy products (77)**	**59 (76.6)**	**59 (76.6)B**	**33 (42.8)b**	**19 (24.6)b**
**Total (180)**	**135 (75)**	**138 (76.6)C**	**75 (41.6)**	**45 (25)c**

*The same small and capital letters in rows indicate significant differences about *p* <0.05.

### Antimicrobial resistance pattern of *Helicobacter pylori*isolates

The susceptibility of *H. pylori* strains to ten commonly used commercial antimicrobial agents was studied in our investigation. Results showed that *H. pylori* strains harbored high levels of antibiotic resistance to ampicillin (84.4%), tetracycline (76.6%), erythromycin (70.5%) and metronidazole (70%) (Table 4). On the other hand, a lower frequency of resistance was observed against levofloxacin (12.7%), furazolidone (13.8%), clarithromycin (17.7%) and cefsulodin (21.1%).

**Table 4:** **Antibiotic resistance of** ***Helicobacter pylori*** **strains isolated from various types of milk and dairy products**

Type of samples (n. positive)	Antibiotic resistance (%)									
	METR5	AM10	CLRT2	ERT5	TE30	AMX10	FZL1	Lev5	TRP25	Cef30
Bovine raw milk (20)	15 (75)	18 (90)	4 (20)	17 (85)	17 (85)	15 (75)	3 (15)	4 (20)	8 (40)	5 (25)
Ovine raw milk (25)	19 (76)	22 (88)	5 (20)	19 (76)	20 (80)	16 (64)	4 (16)	5 (20)	6 (24)	6 (24)
Caprine raw milk (28)	21 (75)	25 (89.2)	6 (21.4)	20 (71.4)	22 (78.5)	19 (67.8)	4 (14.2)	4 (14.2)	10 (35.7)	6 (21.4)
Buffalo raw milk (12)	9 (75)	10 (83.3)	3 (25)	8 (66.6)	9 (75)	9 (75)	2 (16.6)	2 (16.6)	5 (25)	3 (25)
Camel raw milk (8)	6 (75)	8 (100)	1 (12.5)	7 (87.5)	8 (100)	6 (75)	–	–	2 (25)	1 (12.5)
Donkey raw milk (−)	–	–	–	–	–	–	–	–	–	–
**Total milk (103)**	**70 (67.9)B**	83 (80.5)A*	**18 (17.4)a,b**	**71 (68.9)**	**76 (73.7)C**	**65 (63.1)**	**13 (12.6)a,c**	**11 (10.6)a,b,c**	**31 (30)**	**21 (20.3)a**
Traditional cheese (30)	23 (76.6)	26 (86.6)	6 (20)	22 (73.3)	25 (83.3)	21 (70)	6 (30)	5 (16.6)	15 (50)	7 (23.3)
Traditional cream (15)	10 (66.6)	13 (86.6)	3 (20)	11 (73.3)	12 (80)	10 (66.6)	2 (13.3)	2 (3.33)	6 (40)	4 (26.6)
Traditional butter (5)	3 (60)	5 (100)	–	2 (40)	2 (40)	2 (40)	–	–	1 (20)	1 (20)
Traditional ice cream (27)	20 (74.0)	25 (92.5)	5 (18.5)	21 (77.7)	23 (85.1)	19 (70.3)	4 (14.8)	5 (18.5)	8 (29.6)	5 (18.5)
**Total dairy products (77)**	**56 (72.7)C**	**69 (89.6)A**	**14 (18.1)c**	**56 (72.7)**	**62 (80.5)B**	**52 (67.5)**	**12 (15.5)b**	**12 (15.5)a,b,c**	**30 (38.9)**	**17 (22)a**
**Total (180)**	**126 (70)**	**152 (84.4)A**	**32 (17.7)a,c**	**127 (70.5)C**	**138 (76.6)B**	**117 (65)**	**25 (13.8)**	**23 (12.7)a,b,c**	**61 (38.8)**	**38 (21.1)a,b**

METR5: metronidazole (5 μg/disk); AM10: ampicillin (10 μg/disk); CLRT2: clarithromycin (2 μg/disk); ERT5: erythromycin (5 μg/disk); TE30: tetracycline (30 μg/disk); AMX10: amoxicillin (10 μg/disk); FZL1: furazolidone (1 μg/disk); Lev5: levofloxacin (5 μg/disk); TRP25: trimethoprim (25 μg/disk); Cef30: cefsulodin (30 μg/disk).

**The same small and capital letters in rows have significant differences of *p* <0.05.

To the best of our knowledge, the present study is the first investigation about the molecular detection of *vacA*, *cagA*, *iceA* and *oipA* virulence factors of *H. pylori* strains of bovine, ovine, caprine, buffalo and camel milk and their derived dairy products.

Total prevalence of *H. pylori* in bovine, ovine, caprine, buffalo and camel raw milk samples of our survey were 16.66%, 35%, 28%, 15% and 13.33%, respectively. Rahimi and Kheirabadi [5] reported that the incidence of *H. pylori* in raw bovine, ovine, caprine, buffalo and camel milk samples of Iranian herds were 1.41%, 12.2%, 8.7%, 23.4% and 3.6%, respectively. In a study carried out in Italy, *H. pylori* was detected in 50%, 33%, and 25.6% of raw bovine, sheep, and goat milk, respectively [21]. In Japan, a study detected *H. pylori* in 72.2% of raw cow milk samples [22]. Total distribution of *H. pylori* in milk samples of Greek [23] and American [4] herds were 20% and 60%, respectively. Recent clinical investigation among Iranian cows showed that 16% of milk and 40% of feces samples of seropositive herds tested positive for *H. pylori*[6].

Regarding dairy products, 30% of cheese samples were positive for *H. pylori*. The temperature required for cheesemaking is mainly low. In traditional conditions, in order to create a better clot, the average temperature is less than 30°C. In such circumstances and given the low quality of primary milk and unsanitary conditions of dairy products processing, high levels of *H. pylori* contamination are not unimaginable. The transformation of *H. pylori* into coccoid forms may be another explanation for the high incidence of bacteria in the observed conditions that include acidic environment and high temperature.

Substantial discrepancy in the prevalence of *H. pylori* in different studies could be related to variations in the type of tested sample, number of samples, sampling method, experimental methodology, geographical area, and climate differences in the regions where the samples were collected.

There is indirect hypothesis of *H. pylori* transmission through milk, similar to that obtained for water, but less extensive [4, 6, 22–24]. These studies led to the hypothesis that *H. pylori* infection can be considered a zoonosis, which is further reinforced by the occurrence of this bacterium in the gastric mucosa of calves, pigs, and horses and its isolation from gastric tissue and milk of sheep [4]. Such findings suggest that these animal species may act as reservoirs and spreaders of *H. pylori*. Moreover, Momtaz *et al.*[7] showed that *vacA s1a/m1a* was frequently found in *H. pylori* isolated in clinical samples of cow, sheep and humans. They showed 3.4 to 8.4% variability and 92.9 to 98.5% homology between sheep and human samples [7].

Unfortunately, there is no previously published data on the presence of *H. pylori* in dairy products. Among the main reasons for the presence of the bacteria in dairy products are two factors: primary contamination of milk and cross contamination. Use of contaminated water during dairy products processing, handling contamination, use of contaminated equipment and finally lack of public and individual hygiene. Food safety regulations as well as quality standards – including good agricultural practices (GAPs), good manufacturing practices (GMPs), and hazard analysis and critical control points (HACCP) – should be introduced in Iranian food units in order to control contamination and proliferation of pathogenic bacteria.

Close association of *cagA*, *vacA*, *iceA* and *oipA* virulence factors with interleukin 8 (IL-8) production, cytotoxin production, gastric epithelial cells adhesion, inflammatory effects, vacuolization and apoptosis in gastric epithelial cells has been previously observed [25–28]. High prevalence of *cagA*, *vacA*, *iceA* and *oipA* virulence factors in cases of gastritis, peptic ulcer and gastric cancer has been reported in the United States, Turkey, Japan and Brazil [29–32]. Since *H. pylori* isolates in our study harbored *cagA* (76.6%) and *vacA* (75%) genes, consumption of milk and dairy products contaminated with virulent strains may provoke duodenal ulceration, gastric mucosal atrophy and gastric cancer.

Another important finding of our investigation was the high presence of antibiotic resistance among *H. pylori* strains isolated from milk and dairy products. Thyagarajan *et al.*[33] and Secka *et al.*[34] reported that *H. pylori* strains from clinical samples had the highest levels of resistance against metronidazole, amoxicillin, ampicillin and tetracycline, which was similar to our results. In a study conducted in India, resistance of *H. pylori* strains against metronidazole, clarithromycin and amoxicillin were 77.9%, 44.7% and 32.8%, respectively [33]. Similar results were reported by Bang *et al.*[35], who found that *H. pylori* isolates were highly resistant to metronidazole (34.7%), clarithromycin (16.7%) and amoxicillin (11.8%). The low antibiotic resistance of *H. pylori* against levofloxacin, furazolidone, clarithromycin, trimethoprim, and cefsulodin may be due to the less frequent prescription of these antibiotics. A possible explanation for the augmented incidence of antibiotic resistance in our study is the indiscriminate and unnecessary veterinary use of these antibiotics.

## Conclusions

In Iran, milk and dairy products samples were found to harbor *H. pylori* strains that possess the virulence genes *vacA*, *cagA*, *oipA* and *iceA*. Therefore, consumption of raw milk and their derived products by humans may be the source of *H. pylori* infections. Our findings should raise awareness on *H. pylori* resistance to antibiotics in the country. Clinicians should be cautious when prescribing antibiotics, since metronidazole, ampicillin, erythromycin, tetracycline and amoxicillin are not recommended for treating *H. pylori* infections. Based on the present results, we suggest the prescription of clarithromycin, furazolidone, levofloxacin, trimethoprim and cefsulodin.

## Abbreviations

PCR: Polymerase chain reaction
SPSS: Statistical package for the social sciences
*vacA*: Vacuolating cytotoxin
*cagA*: Cytotoxin-associated gene
*iceA*: Induced by contact with epithelium
*oipA*: Outer inflammatory protein.
